# *Drosophila melanogaster* as a Translational Model System to Explore the Impact of Phytochemicals on Human Health

**DOI:** 10.3390/ijms241713365

**Published:** 2023-08-29

**Authors:** Carlos Lopez-Ortiz, Celeste Gracia-Rodriguez, Samantha Belcher, Gerardo Flores-Iga, Amartya Das, Padma Nimmakayala, Nagamani Balagurusamy, Umesh K. Reddy

**Affiliations:** 1 Department of Biology, Gus R. Douglass Institute, West Virginia State University, Institute, WV 25112-1000, USA; carlos.ortiz@wvstateu.edu (C.L.-O.); celeste.rodreguez@wvstateu.edu (C.G.-R.); sbelcher7@wvstateu.edu (S.B.); juan.iga@wvstateu.edu (G.F.-I.); adas@wvstateu.edu (A.D.); padma@wvstateu.edu (P.N.); 2 Laboratorio de Biorremediación, Facultad de Ciencias Biológicas, Universidad Autónoma de Coahuila, Torreón 27275, Coahuila, Mexico; bnagamani@uadec.edu.mx

**Keywords:** *Drosophila*, phytochemical, human health, metabolism, disease

## Abstract

Fruits, vegetables, and spices are natural sources of bioactive phytochemicals, such as polyphenols, carotenoids, flavonoids, curcuminoids, terpenoids, and capsaicinoids, possessing multiple health benefits and relatively low toxicity. These compounds found in the diet play a central role in organism development and fitness. Given the complexity of the whole-body response to dietary changes, invertebrate model organisms can be valuable tools to examine the interplay between genes, signaling pathways, and metabolism. *Drosophila melanogaster*, an invertebrate model with its extensively studied genome, has more than 70% gene homology to humans and has been used as a model system in biological studies for a long time. The notable advantages of *Drosophila* as a model system, such as their low maintenance cost, high reproductive rate, short generation time and lifespan, and the high similarity of metabolic pathways between *Drosophila* and mammals, have encouraged the use of *Drosophila* in the context of screening and evaluating the impact of phytochemicals present in the diet. Here, we review the benefits of *Drosophila* as a model system for use in the study of phytochemical ingestion and describe the previously reported effects of phytochemical consumption in *Drosophila*.

## 1. Introduction

Phytochemicals are specialized metabolites with biological properties stored in plant tissues, such as roots, stems, leaves, flowers, and fruits [1]. Phytochemicals include a wide range of compounds such as polyphenols, carotenoids, flavonoids, coumarins, terpenoids, glucosinolates, saponins, and capsaicinoids, which are often associated with the vibrant colors of fruits and vegetables [2]. Although phytochemicals are not essential nutrients in plants, they are responsible for many health benefits associated with a plant-based diet [3,4]. Potential phytochemical health benefits include antioxidant, anti-inflammatory, anti-cancer, and anti-microbial properties [5]. Currently, numerous phytochemicals are being studied for their possible use in developing novel drugs and dietary supplements [6].

Phytochemicals exhibit their beneficial and harmful effects by interaction with multiple cell signaling molecules [7]. Nevertheless, the molecular mechanisms underlying the effectiveness of phytochemicals continue to grow. In this context, employing cost-effective, rapid, reliable, and efficient in vitro and in vivo assays will facilitate analyzing these compounds’ metabolic processes, dosage response, and pharmacological and toxicological profiles [8,9,10]. *D. melanogaster*, known as the fruit fly, has emerged as an alternate animal model. Due to its short lifespan, small size, and well-understood genome, it has been widely used to examine the efficacy and safety of phytochemicals on various physiological processes, including metabolism, aging, and immunity [11,12].

Moreover, the central nervous system of *Drosophila* allows the study of diverse functions, including special senses, such as olfaction, taste, hearing, and vision, as well as motor behavior, including flight, walking, learning, and memory in response to phytochemical consumption [13,14]. Likewise, *Drosophila* has been shown to play a crucial role in deciphering fundamental molecular mechanisms and aiding in developing phytochemical base drugs for cancer and neurodegenerative diseases, such as Alzheimer’s and Parkinson’s, and their associated genes [15,16,17,18]. In this chapter, we provide a brief overview of the effectiveness of *Drosophila* as an alternative model organism for evaluating the impact of phytochemicals on human diseases.

## 2. Phytochemicals and Their Potential Therapeutic Benefits

Regular consumption of fruits, vegetables, and grains has been associated with a reduced risk of certain chronic diseases due to phytochemicals with antioxidant and anti-inflammatory properties (Table 1) [19]. Phytochemicals regulate oxidative stress, which has been recognized as a significant factor in the pathogenesis of metabolic disorders and cancer [20]. Phytochemical therapeutic benefits are divided mainly into five categories (i) enhancers of the body’s immune system; (ii) preventers of diabetes and heart diseases; (iii) hypocholesterolemic agents; (iv) promoters of digestion and absorption; and (v) retardants of aging [21,22]. The major classes of phytochemicals with disease-preventing functions are dietary fiber, antioxidants, detoxifying agents, immunity-potentiating, and neuropharmacological agents [23].

For instance, polyphenols such as resveratrol have been associated with decreased risk of myocardial infarction, stroke, and diabetes [24,25]. Polyphenols in diet also help to improve lipid profiles, blood pressure, insulin resistance, and systemic inflammation [26]. Furthermore, vitamin C and carotenoids may benefit immune function, thereby reducing cancer risk by enhancing the tumor surveillance of the immune system [27]. Capsaicinoids, including capsaicin and dihydrocapsaicin found in peppers, have been found to have beneficial roles in humans, including managing pain inflammation during rheumatoid arthritis, anti-cancer agent by generating reactive oxygen species, and in the prevention or treatment of neurodegenerative diseases such as Alzheimer’s disease due to its antioxidant activity [28,29,30]. Likewise, carotenoids and capsaicin have been found to have anti-obesity effects during dietary consumption by promoting fatty acid oxidation and regulating appetite and satiety, respectively [31,32]. Moreover, it has been shown that intake of carotenoids besides β-carotene, such as lutein, zeaxanthin, and lycopene, resulted in elevated levels of blood carotenoids related to a reduced risk of lung cancer [33,34].

Other spices, such as turmeric obtained from the roots of *Curcuma domestica*, contain a yellow coloring principle, curcumin, a powerful antioxidant that can offer protection against cancer, inhibiting lipid peroxide-induced DNA damage [35]. Flavonoid consumption, such as quercetin and kaemferol, through vegetables and fruits, reduces the risk of death from coronary heart disease [36]. Besides the chemicals with specific functions in plant food, plant foods also contain many other chemical compounds, such as acids, esters, bases, and phenolic compounds [37]. It is unclear whether these compounds also have any beneficial biological function in the body. They may have a role in stimulating appetite and satiety [38].

## 3. Advantages of Using *Drosophila* as a Translational Model for Testing Phytochemicals

For decades, *D. melanogaster* has been widely used as an excellent animal model to study genetics, evolution, and developmental biology [39]. It is a cost-effective option due to its high reproductive rate (30–50 eggs/day), short generation time (approximately ten days at 25 °C), and low maintenance cost. In addition, *Drosophila* short lifespan (average three months at 25 °C) and easy generation of large populations allow for performing longevity and lifespan assays in only a few months [40]. Furthermore, it offers powerful molecular and genetic tools that permit gene overexpression or knock-down studies [41].

Although *Drosophila* is evolutionarily distant from humans, fly development, physiological, biological, and metabolic processes are equivalent to many of those found in mammals. Recently, similarities between humans and fruit flies in terms of metabolic regulation, including the role of insulin signaling, nutrient sensing, and energy homeostasis in metabolic disorders, such as diabetes and obesity [42,43,44,45]. Moreover, ingesting complex foods rich in phytochemicals by an organism leads to the degradation of nutrients that directly affect the gastrointestinal microbiome because the host and microbiome share the same food source [46]. These microbiome changes influence the organism’s phenotype and behavior by altering the genome, transcriptome, epigenome, proteome, and metabolome [47].

Furthermore, the similarities between pathological mechanisms of diseases in flies and humans and the ease of genetic manipulation of the fly make *Drosophila* suitable as a primary model for the study of phytochemical effects in neurodegenerative diseases, such as Alzheimer’s disease, Parkinson’s disease, Huntington’s disease, cardiovascular disease, muscular atrophy, aging, and metabolic diseases [48,49,50]. These advantages of *Drosophila* are even more evident in the field of research into plant-based drug discovery. As it allows whole-organism screening, using a *Drosophila* model for nutraceutical effects has distinct advantages over cell-based assays when investigating more complex phenotypes [51]. To date, *Drosophila* has been used as a model for in vivo screening of candidate plant-derived compounds for use in age-neurodegenerative-related diseases and metabolic disorders to investigate the action mechanisms of phytochemicals with therapeutic potential [52].

## 4. Methods for Testing the Efficacy of Phytochemicals in *Drosophila*

Phytochemical ingestion effects are observed in general physiology, including metabolism, behavior, stress resistance, reproductive capacity, nervous system, and immune capacity in both *Drosophila* and humans, and aspects of these physiological changes can be used as parameters to determine the phytochemical effects and toxicity [50]. Among them, lifespan and survival rate are simple and efficient longitudinal assays to determine the effects after administration of the candidate plant-based compounds [53]. In addition, various strains with different lifespan characteristics and transgenic flies with symptoms similar to human diseases are available to evaluate plant compounds’ effects on mortality rate. Notably, the direct and indirect effects of phytochemical compounds on mortality rate should be determined since mortality rate can be affected by several physiological confounders such as fecundity, metabolic rate, and amount of food administered [54]. In addition to establishing the mortality rate, determining changes in fly motility by quantifying the ability to climb (negative geotaxis) and reproductive output is popular when assessing the health span of *Drosophila* [55].

**Figure 1:**
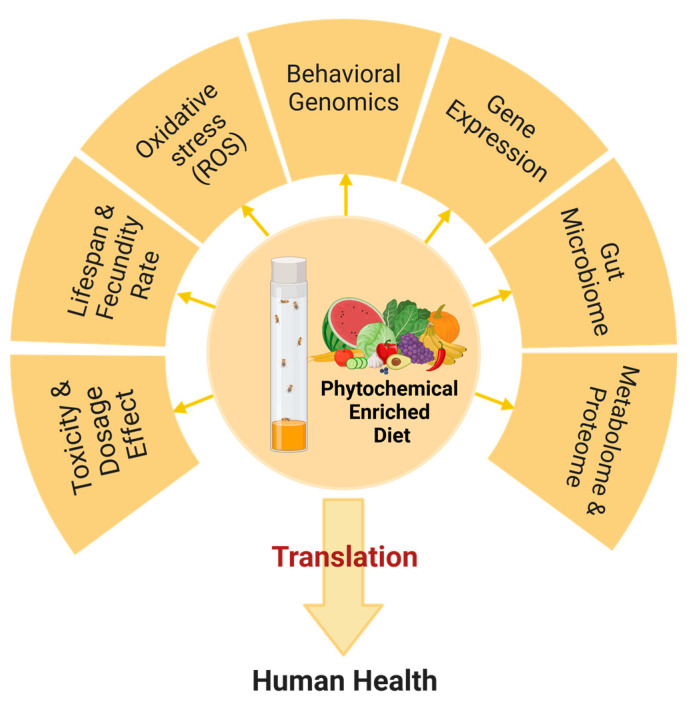
Approaches for evaluating the effects of phytochemicals in *Drosophila melanogaster* model.

Oxidative stress mitigation in *Drosophila* is also an essential parameter in assessing the efficacy of phytochemicals. Levels of reactive oxygen species (ROS), activities of antioxidant enzymes, such as superoxide dismutase and catalase, and lipid peroxidation in treated fly cells provide insight into the potential antioxidative activities of phytochemicals (Figure 1) [56]. Moreover, *Drosophila* has a relatively simple nervous system, making it an ideal model to study neurodevelopment and neurodegeneration using automated tracking systems, locomotor activity, ring assay, gustatory, social, and circadian rhythm patterns, providing insight into potential impacts of phytochemicals on the nervous system function and behavior [57]. Further, omics technologies, including transcriptomics and metabolomics, are also applied to *Drosophila*, allowing for a more global survey of the regulation of genes and metabolites, resulting in even more fine-grained analyses and deeper insights [58]. Using omics technologies, for example, the differential metabolic and gene expression of female and male flies can be analyzed. In addition, special genetic tools, such as RNA interference and CRISPR/Cas9 gene editing, can be used to investigate the biological function of genes that may be up or down-regulated in response to phytochemical consumption.

Although administration of compounds via feeding is the most typical method of phytochemical delivery to *Drosophila* [50], it is important to consider several concerns about feeding-related artifacts, such as reduced feeding related to the preference of flies for each compound, uncertainty about the amount of food consumed by each individual and of the actual phytochemical concentration achieved in the tissues of the flies. Thus, it is necessary to measure the food amount ingested by the flies, which can be done by several methods, such as using food colorant or capillary feeding assays to monitor food intake [59]. Various phytochemical concentrations should also be tested to evaluate dose responses and outcomes controlling supplementation timing and diet composition.

## 5. Studies Evaluating the Effect of Phytochemicals by Using *Drosophila*

### 5.1. Phytochemical Effect on Aging

Anti-aging research has gained significant attention due to the growing aging population, which experiences a natural decline in the body’s capacity to repair molecular, biochemical, and organ damage, resulting in increased susceptibility to age-related diseases [60]. The primary cause of radical damage to macromolecules in aging is the progressive decline of the endogenous antioxidant system, triggered by reactive oxygen species (ROS). To restore the ROS balance, natural plant-derived antioxidants can be utilized to reinforce the endogenous antioxidant system [61].

Plant-derived compounds with anti-aging properties have been investigated. *Moringa oleifera* has been identified as one of the plants with the highest levels of bioactive molecules, such as polyphenols, flavonoids, and tannins, which elicit an antioxidant response [62]. Iorjiim et al. [63] reported a significant increase in the activity of antioxidant enzymes glutathione-S-transferase and catalase in *Drosophila* treated with *M. oleifera* extract, suggesting its role in enhancing the activity of antioxidant enzymes and reducing the harmful effects of ROS on aging. Likewise, when leaf extracts of *M. oleifera* were administered to *Drosophila* at a dose of 5 mg·mL^−1^ as a dietary supplement, the flies exhibited a remarkable increase in lifespan compared to the control group. The survival rate of the treated *Drosophila* was extended by 20 days, and their mobility and climbing activities were also enhanced, indicating a positive impact on aging and survival. However, concentrations exceeding 2000 mg·mL^−1^ increase the risk of fly deterioration caused by toxic effects [64].

Withanolides are widely found in *Solanaceae* species and have been investigated for their potential to enhance resilience against age-related stress [65]. *Withania somnifera* extracts with high withanolides concentration have been shown to ameliorate behavioral deficits in an in vivo *D*. *melanogaster* model of oxidative stress, reducing the effects of aging in locomotion and cognition [66]. In addition, *W. somnifera* extracts have been reported to improve physical fitness and alleviate age-related sleep fragmentation [67].

### 5.2. Development and Lifespan

**Figure 2:**
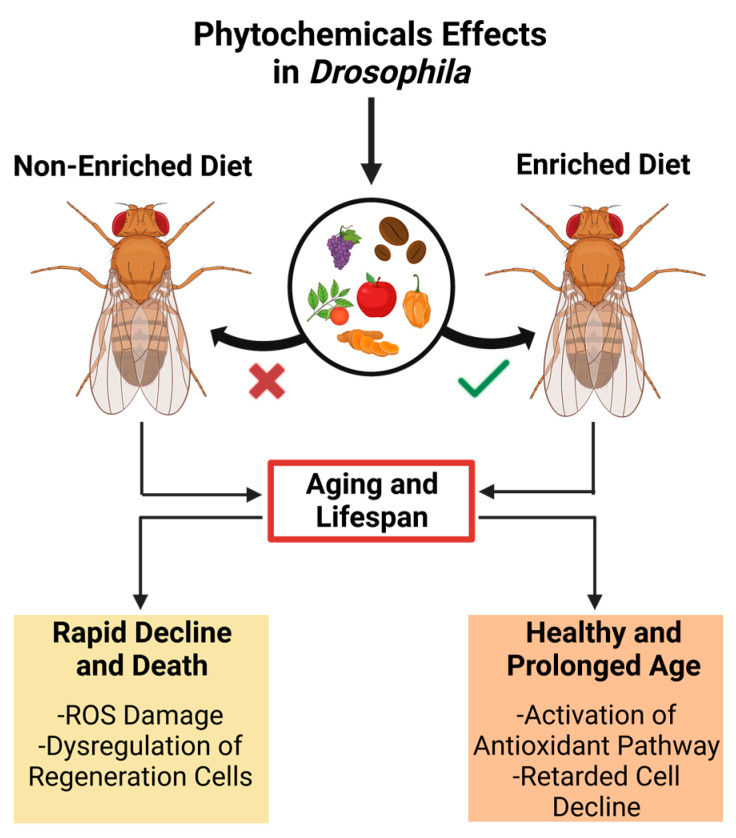
The impact of a phytochemical-enriched diet on the lifespan of *Drosophila melanogaster* suggests potential applications for promoting healthy aging and longevity in humans.

Age-related dysregulation of development is closely associated with functional organ and tissue decline, affecting lifespan and age-related disease development [60]. A modern lifestyle characterized by high caloric intake and minimal physical activity in humans results in high lipid storage levels that reduce overall lifespan [68]. Nevertheless, several studies have shown that several phytochemicals can expand the lifespan using *Drosophila* as a model (Figure 2) [69].

For instance, in the *Drosophila* midgut model, phenolic caffeic has been shown to slow down the decline of intestinal functions in aged *Drosophila*, preventing dysregulation of regeneration and differentiation cells during aging and thus increasing lifespan [70]. Moreover, Li et al. [71] reported that catechins, commonly found in foods such as cacao and edible plants such as green tea and red wine, can decrease mortality rates and prolong the lifespan of flies reared on a high-fat diet when supplemented with a 10 mg·mL^−1^ extract of green tea containing 62% epigallocatechin gallate and 19% of epicatechin gallate. Similarly, it has been reported that epigallocatechin gallate affects glucose metabolism and upregulates superoxide dismutase and catalase enzymatic activities in fruit flies, increasing fitness and lifespan [71,72].

Likewise, curcumin is an effective inhibitor of inflammation and oxygen radicals [73]. It has been shown that fruit flies maintained on a media containing 1.0 mg of curcumin per gram had a higher mean lifespan, linking this effect to the enhancement of superoxide dismutase activity [74,75]. In addition to the previously mentioned compounds, other phytochemicals, such as morphine, an opiate analgesic extracted from opium poppies, have been recognized as a significant signaling molecule. Despite its undesirable effects, such as respiratory depression and physical dependence, morphine has been identified as crucial in protecting against post-traumatic stress. Additionally, it stimulates the growth and regeneration of nervous fibers and has also been found to increase the lifespan of *Drosophila* flies [76]. In a study, male and female Oregon-R flies were given morphine hydrochloride once a week at a dosage range of 0.001–0.25 mg·mL^−1^, which resulted in an extended lifespan [50].

### 5.3. Metabolism

Recent evidence suggests that the metabolic state of an organism is closely tied to its diet, with an important factor being that the dietary habits of parents can impact the metabolic states of their offspring [77]. Controlled dietary conditions are crucial for studying metabolism and organism physiology [78]. *Drosophila* possesses notable metabolic systems that share many conserved functions with vertebrates, including insulin, insulin-like growth factor, the target of rapamycin signaling pathways, and energy regulation [79,80].

Obesity is a prevalent metabolic syndrome in humans, leading to various metabolic complications, such as impaired glucose tolerance, insulin resistance, dyslipidemia, hypertension, type 2 diabetes, and premature heart disease [81]. Heinrichsen et al. [82] evaluated the metabolic response of *Drosophila* fed a high-fat diet, which resulted in increased levels of triglyceride and glucose, decreased stress tolerance and lifespan, and activation of pathways associated with fat metabolism, insulin signaling, cardiac fat accumulation, and dysfunction. Currently, the options for treating obesity and implementing lifestyle interventions are limited, making it challenging to maintain a healthy lifestyle [83]. To address this, researchers have explored the potential of various phytochemicals for their anti-obesity properties [84].

Recently, it was reported that flies reared on a habanero pepper-supplemented diet with high capsaicin and carotenoid content showed a significant body weight and triglyceride reduction compared to those fed with the control diet [31]. Carotenoids and capsaicin in pepper have been reported to have anti-obesity effects when consumed in the diet, promoting fatty acid oxidation and regulating appetite and satiety, respectively [85,86].

Moreover, the effects of resveratrol were also analyzed in *Drosophila* metabolism. It was observed that resveratrol supplementation improved metabolic parameters, such as enhanced glucose tolerance and reduced lipid accumulation. Nevertheless, the implementation of target-specific therapy might be beneficial in mitigating any negative consequences arising from the pro-oxidant activity associated with high dosages [87]. Meanwhile, quercetin supplementation, a flavonoid in various fruits and vegetables, improved glucose homeostasis, reduced oxidative stress, and enhanced mitochondrial function in flies [88]. Further, garlic and purple onion were tested for their biological properties in metabolic disorders. Combined as a diet, these vegetables significantly ameliorated total glucose and triglyceride levels, surpassing the effects of consuming each vegetable individually. This suggests that the combination of garlic and purple onion may possess antihyperglycemic properties [89]. Likewise, it was reported that radish sprouts (*Raphanus sativus* cv. Rambo), a *Brassicaceae* species rich in glucosinolates, influenced energy metabolism in *Drosophila*, leading to lower glucose levels and altered the expression of the insulin signaling-related gene called “*spargel*” [90]. Leaf and root extracts have also been used to treat Diabetes mellitus, the most common human metabolic disorder [91]. For instance, *Drosophila* flies treated with *Senna occidentalis* and *Artocarpus camansi* leaf extracts showed a significant decrease in serum glucose levels and antioxidant properties that mediate hyperglycemia in diabetes compared to the control flies [92,93].

### 5.4. Microbiome

The gut microbiome in the human intestines plays a critical role in nutrient absorption, lysis, and storage [94]. Additionally, they are essential for various physiological processes, including metabolism, digestion, circadian rhythms, and vitamin synthesis in humans [95]. Imbalances in the gut microbiota (dysbiosis) due to diet, antibiotic use, age, and stress contribute to disease development, including diabetes, obesity, colon cancer, inflammatory bowel disease, inflammation, and neurodegeneration [96].

**Figure 3:**
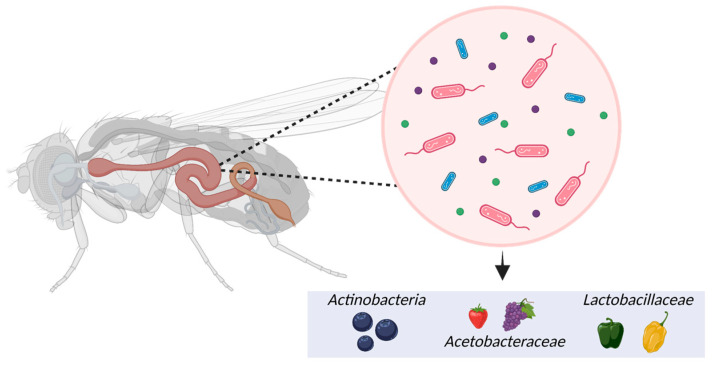
Impact of phytochemicals on the microbiome of *Drosophila melanogaster*.

The *Drosophila* gut microbiome is extracellular and encompasses three regions: the foregut, midgut, and hindgut, each creating distinct conditions for microbial cells [97]. Although the *Drosophila* gut microbiome has been well-documented, it is important to note that fruit flies have a limited number of microorganisms, about 30 species, compared to mammals with more than 500 species [98]. The gut microbial population is profoundly influenced by the dietary habits associated with consuming different types and amounts of phytochemicals (99). It also varies depending on the host genotype, age, sex, and habitat [99,100]. Garcia-Lozano et al. [101] conducted a study using three different *Drosophila* genotypes (Berlin-K, Oregon-RC, and Canton-s) and three distinct diets supplemented with bell, serrano, and habanero peppers, respectively. The results showed that pepper-containing diets appeared to enhance members of *Lactobacillaceae* and *Acetobacteraceae* in the *Drosophila* gut microbiome (Figure 3). Among them, *L. brevis* belonging to the *Lactobacillaceae* family was 4-fold higher in flies reared on pepper-containing diets than in the guts of flies raised on the control diet. *L. brevis* is traditionally consumed for its prophylactic and therapeutic benefits against various diseases, such as human inflammatory bowel syndrome [102].

Moreover, Jimenez-Padilla et al. [103] found that *Drosophila* flies fed diets with strawberries and blueberries had an increased abundance of *Acetobacter* in their microbiome. *Acetobacter*, particularly *Acetobacter pomorum*, has probiotic properties and produces acetic acid, which promotes insulin signaling, reducing lipid and sugar levels in adult flies [104]. The blueberry diet also led to higher levels of *Actinobacteria* compared to the control diet [103]. *Actinobacteria*, including the genus *Bifidobacterium*, metabolize anthocyanins into small compounds with probiotic effects for obesity and gastrointestinal and systemic diseases [105,106].

Furthermore, it has been reported that Triphala, a novel polyphenol-rich prebiotic at 0.5% *w/v* concentration, supports the growth of beneficial bacteria such as *Lactobacillus plantarum*, *Lactobacillus fermentum,* and *Bifidobacteria infantis* while inhibiting pathogenic species by using a simulated model of the human gastrointestinal tract (SHIME) in *D. melanogaster* [107].

### 5.5. Neurodegenerative Diseases

*D. melanogaster* has been widely used in drug screening studies to identify high-quality hits that exhibit crucial characteristics, including metabolic stability, oral or transdermal availability, and, most notably, low toxicity, providing a valuable resource for drug development [40]. Phytochemicals have been extensively studied for their potential in preventing and controlling the proliferation and development of tumor cells [108]. Black beans, specifically *Phaseolus vulgaris*, contain phenolic compounds, including cyanidin-3-O-glucoside (C3G), recognized as an anti-cancer compound [109]. A recent study by Wei et al. [110] investigated the effects of an extract from the black bean seed coat on a *Drosophila* model with an activated oncogene *Raf* (Table 2). It was observed that flies treated with black bean extract exhibited a significant reduction in tumor proliferation and a blockage of autophagy in the tumor cells.

Likewise, phytochemicals can offer neuroprotection, which is crucial for developing new treatments for neurodegenerative diseases, such as Alzheimer’s. In Alzheimer’s disease, memory loss is associated with the forming beta-amyloid plaques, which give rise to oligomers that generate reactive oxygen species (ROS) and promote Tau protein aggregation, ultimately leading to neuronal cell death [111]. Studies have shown that extracts from *Pueraria tuberosa* can inhibit beta-site amyloid precursor protein cleaving enzyme (*BACE1*), a key target in forming beta-amyloid plaques [112]. Significant improvements in cognitive decline were observed in *Drosophila* models treated with these extracts, whereas in humans, adverse effects such as nausea and vomiting have been observed [113]. Additionally, extracts rich in limonene (+) have demonstrated a neuroprotective effect by reducing cell death in treated *Drosophila* and lowering brain ROS levels and inflammation [111].

Similarly, studies have shown that plant-derived molecules can be effective in treating Parkinson’s disease (PD), which is characterized by the formation of Lewy bodies containing alpha-synuclein and the loss of dopaminergic neurons resulting in motor symptoms [114]. Gardenine-A, a phytochemical derived from the plant *Gardenia resinifera*, has been found to have neuroprotective effects in *Drosophila* models of PD by reducing mortality and modulating inflammatory and cellular responses [115]. In another study, lemongrass citral extract was tested in silico and in vivo in *Drosophila* models of PD, showing enhanced climbing ability. It reduced ROS levels, with positive interactions observed between citral and alpha-synuclein in molecular docking analyses [116].

Moreover, epilepsy is a neurological disorder characterized by sensory-motor deficits and convulsions. It can have various causes, including gene mutations that encode ion channels in brain cells responsible for transmitting signals between neurons [117]. To alleviate epilepsy symptoms, researchers have investigated using *Imperata cylindrica* root extracts in a mutant *Drosophila* “*para*” gene model. The treated flies exhibited an inhibitory effect on voltage-gated sodium ion channels, which reduced inflammation and increased tissue repair in brain cells, confirming the extract’s efficacy in treating epilepsy [117].

## 6. Gene Regulation Induced by Phytochemicals in *Drosophila*

**Figure 4:**
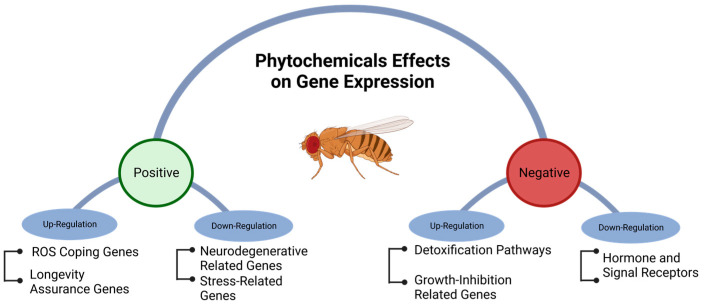
Positive and negative genetic regulation of metabolic pathways in *Drosophila melanogaster* exposed to phytochemicals.

Phytochemical intake can modulate gene expression, influenced by factors, such as cell type, life stage, and growing conditions. Different mechanisms of gene regulation, including cis-regulatory elements, repressor proteins, non-coding RNA, and epigenetic processes, such as methylation, may be affected by phytochemicals [93,118,119,120]. High-throughput technologies, such as RNA-seq, are commonly used to study gene regulation and metabolic pathways on a genome-wide scale [120,121]. Phytochemicals have the potential to modulate metabolic pathways and coping mechanisms that counteract age-related neurodegenerative diseases [122]. Although phytochemicals can positively influence the health of *D. melanogaster* by upregulating genes associated with longer lifespans and down-regulating genes related to diseases and stress, they can also have negative effects by upregulating genes associated with reduced growth rates. Additionally, they may trigger mechanisms related to detoxification and reduce the expression of genes involved in coping with reactive oxygen species (ROS) and hormone-signaling receptors (Figure 4).

A study by Lee et al. [75] revealed that supplementing *Drosophila* with curcumin can influence the expression of aging-related genes such as *mth*, *thor*, *InR*, and *JNK*. These genes are associated with the insulin, JNK, and methuselah signaling pathways. The modulation of gene expression by curcumin leads to a delay in the onset of age-associated gene expression and an increase in the lifespan of *Drosophila* flies. Likewise, Zhang et al. [121] have reported that curcumin can augment the activities of the Notch and Wnt signaling pathways, leading to the disruption of the cell division cycle, specifically in cells harboring DNA damage. Nevertheless, flies fed *Piper nigrum* extract-supplemented diets showed reduced activity of the *Tom* gene (*CG5185*), part of the Notch signaling pathway. *Piper nigrum* extracts contain active compounds, such as 4,5-dihydropiperlonguminine and piperine, which interact with sodium channels, leading to sustained neuronal activation. However, these compounds also inhibit polysubstrate monooxygenase, potentially slowing metabolism in *Drosophila* [123].

Moreover, Adedara et al. [124] reported that resveratrol supplementation in flies with *parkin* gene mutations resulted in the upregulation of *ple* and *Sod1* genes involved in dopamine biosynthesis and counteracting free oxygen radicals, respectively. The upregulation of these genes, especially *ple*, is significant in preventing Parkinson’s disease, associated with the depletion of dopamine levels and aging. However, Staats et al. [125] found that resveratrol did not affect the expression of other stress-related genes, such as catalase or longevity assurance genes, such as sirtuin (*Sir2*) and spargel (*srl*).

In a recent study, Lopez-Ortiz et al. [31] examined the transcriptional responses to a habanero-pepper diet in the *Drosophila* fly model. Five hundred thirty-nine genes were differentially expressed in flies fed a pepper versus a control diet. Transcriptome results indicated that genes were upregulated, including gustatory receptors and odorant-binding proteins involved in olfactory perception and nutrient processing. In contrast, some stress response-related genes were down-regulated. In addition, *Muc68Ca*, *Muc30E*, and *CG2839*, orthologs of human *Muc2* and *Reg3g*, respectively, were upregulated. These genes are known to play a protective role and regulate hormone secretion in the digestive system [126,127]. Pepper consumption also upregulated an adipokinetic hormone, *Akh*, principally known for its mobilization of energy substrates, triggering the conversion of stored glycogen and lipids to free energy through triglyceride breakdown [128]. Moreover, the gene inactive (*iav*), which is an orthologue of *TRPV1* (transient receptor potential cation channel subfamily V member 1) in humans in response to capsaicin and has been reported to promote chemoresistance in non-small-cell lung cancer [129]. In *Drosophila*, *iav* is involved in several processes, including adult walking behavior, negative gravitaxis, and sensory perception of mechanical stimuli, such as the sensation of heat caused by capsaicin from habanero peppers [130]. Overall, these findings suggest that the consumption of pepper-containing phytochemicals such as capsaicin and carotenoids can lead to altered perception and behavior in *Drosophila*, as well as an impact on nutrient sensitivity and fat oxidation metabolism.

## 7. Challenges and Limitations of Using *Drosophila* as a Translational Model

The significant genetic and physiological differences between *Drosophila* and humans and the additional layers of regulation and organ systems in humans hinder the direct translation of phytochemical consumption observed responses in flies. Likewise, metabolic and pharmacokinetic variations between *Drosophila* and humans can lead to differences in exposure levels and bioavailability of phytochemicals, affecting the observed effects and limiting the translation of dosage and treatment regimens. Moreover, the complex nature of human diseases, involving multiple factors, makes it challenging for *Drosophila* models to fully capture disease mechanisms and reflect the intricacy of human diseases. To overcome these limitations, it is essential to integrate findings from multiple model systems, starting from the *Drosophila* model, including in vitro studies in rats and human clinical trials. This comprehensive approach will help bridge the gap between *Drosophila* research and translational applications in human health, providing more reliable evidence regarding the potential benefits and risks associated with phytochemical use in humans.

## 8. Future Directions and Opportunities for Using *Drosophila* to Test Phytochemicals

*D. melanogaster* has proved to be an invaluable model organism for investigating the impact of phytochemicals on various biological processes. Future studies can further our understanding of the underlying molecular mechanisms that drive these effects, including identifying specific target genes, signaling pathways, and molecular interactions involved in mediating observed phytochemical effects. In addition, comparative analyses across different *Drosophila* strains or species can provide information on the genetic variations contributing to differential responses to phytochemical ingestion. It is also essential to investigate the long-term effects of phytochemical exposure, including transgenerational and age-related outcomes, to assess the potential benefits or risks associated with prolonged phytochemical consumption.

## 9. Conclusions

Plant extracts have long been employed for their therapeutic and preventive properties in addressing various disorders. These extracts encompass a wide array of bioactive compounds, including polyphenols, carotenoids, flavonoids, curcuminoids, terpenoids, and capsaicinoids, contributing to their potential beneficial effects. *Drosophila* is an excellent model organism with extensive use in studying diverse biological processes. Leveraging the vast range of powerful genetic and molecular biology tools available, the *Drosophila* model offers a valuable and cheap alternative for investigating the effects of plant extracts and their derived compounds on large populations in a short period.

## Figures and Tables

**Table 1:** Source of phytochemicals in plants and fruits and their potential beneficial activity against human diseases.

Plant/Fruit	Phytochemicals	Description	Beneficial Activity in Human Related Disease
Pepper	Capsaicinoids Carotenoids	Promote fatty acid oxidation and antioxidant activity	Obesity
*Curcuma* *domestica*	Curcumin	Antioxidant, inhibition of lipid peroxide-induce DNA damage	Cancer Lifespan
*Moringa* *oleifera*	Polyphenols Flavonoids Tannins	Antioxidant response by glutathione-S-transferase and catalase	Aging Lifespan
*Withania somnifera*	Withanolides	Reduces oxidative stress	Aging
*Coffee*	Phenolic caffeic	Prevent dysregulation of regeneration and differentiation cells	Lifespan
*Camellia sinensis*	Catechins	Upregulates superoxide dismutase and catalase	Lifespan
*Papaver* *somniferum*	Morphine	Stimulate growth and nervous fibers	Lifespan
Red grapes	Resveratrol	Antioxidant activity reduces lipid accumulation	Obesity
Radish sprouts	Glucosinolates	Influence energy metabolism and the expression of insulin signaling gene	Diabetes
*Phaseolus vulgaris*	Cyanidin-3-O-glucoside	Reduces tumor proliferation and blocks autophagy	Cancer
*Pueraria tuberosa*	Puerarin	Inhibit the development of beta-amyloid plaques	Alzheimer
*Lemongrass*	Flavonoids Tannins Alkaloids Glycosides	Decreases ROS levels	Parkinson
*Imperata* *cylindrica*	Alkaloids Polyphenolic compounds	Inhibition of voltage-gated sodium channels and reduce inflammation	Epilepsy

**Table 2:** The effects of phytochemicals on genes associated with degenerative diseases.

Gene ID	Gene Name	Annotation	Human Orthologue	Disease	Plant Extract
FBgn0003079	*Raf*	Encodes a serine-threonine protein kinase; it activates the MEK/ERK pathway to regulate cell proliferation.	*Raf-1*	Cancer	*Phaseolus vulgaris*
FBgn0032049	*Bace*	Beta-site APP-cleaving enzyme encodes an aspartic protease that cleaves amyloid precursor proteins.	*BACE1*	Alzheimer	*Pueraria tuberosa*
FBgn0026420	*SNCA*	Engineered foreign gene involves several processes, including negative and positive transport regulation and protein metabolic process.	*SNCA*	Parkinson	*Lemongrass*
FBgn0285944	*para*	A gene is required for locomotor activity. It encodes an α-subunit of voltage-gated sodium channels.	*SCN*	Epilepsy	*Imperata* *cylindrica*
